# Antifungal Potential of *Bacillus* spp., *Streptomyces* spp. and *Trichoderma asperellum* Against Phytopathogenic Fungi

**DOI:** 10.3390/pathogens15050458

**Published:** 2026-04-23

**Authors:** Māris Seņkovs, Vizma Nikolajeva, Luīze Rubene, Kristians Jauga, Līga Zemeca, Inta Jakobija

**Affiliations:** 1 Microbial Strain Collection of Latvia, MIRRI-ERIC Consortium, Faculty of Medicine and Life Sciences, University of Latvia, Jelgavas Street 1, LV-1004 Riga, Latvia; vizma.nikolajeva@lu.lv (V.N.); lr19115@students.lu.lv (L.R.); jauga.kristians@gmail.com (K.J.); 2 Institute of Plant Protection Research “Agrihorts”, Latvia University of Life Sciences and Technologies, Paula Lejiņa Street 2, LV-3004 Jelgava, Latvia; liga.zemeca@lbtu.lv (L.Z.); inta.jakobija@lbtu.lv (I.J.)

**Keywords:** *Fusarium*, *Streptomyces*, *Bacillus*, *Trichoderma asperellum*, *Colletotrichum salicis*, antifungal

## Abstract

The increasing demand for sustainable plant protection products has intensified interest in microbial biocontrol agents (BCAs). This study aimed to evaluate the antifungal activity of selected *Streptomyces*, *Bacillus*, and *Trichoderma asperellum* strains against phytopathogenic fungi and to assess their potential as BCAs under *in vitro* conditions. The antifungal activity of ten *Streptomyces* strains was first evaluated against *Botrytis cinerea*, *Colletotrichum salicis*, *Fusarium oxysporum*, and *F. graminearum* using a dual-culture assay. All isolates exhibited antifungal activity, with *Streptomyces venezuelae* MSCL 350 showing the strongest inhibition. In addition, the antifungal activity of *T. asperellum* MSCL 309 and three *Bacillus* strains was assessed against twelve *Fusarium* spp. isolates obtained from oats. *T. asperellum* demonstrated broad-spectrum inhibition, with growth inhibition ranging from 44.6% to 78.4%, primarily due to soluble metabolites, while volatile compounds showed no significant effect. Among the other tested *Bacillus* strains, only *Bacillus subtilis* MSCL 1441 exhibited antifungal activity, inhibiting all tested isolates. These results demonstrate strong strain-dependent antifungal activity and highlight *T. asperellum* MSCL 309, *S. venezuelae* MSCL 350, and *B. subtilis* MSCL 1441 as promising candidates for the development of environmentally friendly biocontrol agents.

## 1. Introduction

Phytopathogenic fungi represent one of the major constraints to global crop production, causing significant yield losses and compromising food safety. Among them, the genus *Fusarium* is particularly important due to its wide distribution in soils and plant residues and its ability to infect a broad range of agricultural crops. Species such as *Fusarium oxysporum* and *F. solani* are responsible for vascular wilt, root rot, and seedling diseases in cereals, vegetables, and fruit crops [1,2]. *F. graminearum* is a causal agent of *Fusarium* head blight (FHB) of wheat, barley, and other small-grain cereals worldwide [3]; however, other species are also involved. Over the past two decades, the most common species causing FHB in Canada were *F. avenaceum*, *F. equiseti*, *F. graminearum*, *F. poae*, and *F. sporotrichioides*. In Latvia, *F. avenaceum*, *F. culmorum*, *F. graminearum*, *F. poae*, and *F. sporotrichioides* [4] have been identified in populations causing FHB in both spring barley and oat [5].

*Fusarium* pathogenicity is associated with the secretion of cell wall-degrading enzymes, toxins, and effector molecules that facilitate host penetration, tissue maceration, and systemic colonization [6,7]. In addition to direct plant damage, many *Fusarium* species produce mycotoxins—including trichothecenes, zearalenone, and fumonisins—that pose serious risks to animal and human health [8,9]. *F. graminearum* and *F. oxysporum*, together with *Botrytis cinerea* and *Colletotrichum* spp., are ranked among the “top 10” most important fungal plant pathogens based on their scientific and economic impact on agriculture [10], affecting major crops such as cereals, fruits, and vegetables. The World Health Organization has also included *Fusarium* species among high-priority fungal pathogens due to their ability to cause invasive infections in humans and their potential resistance to antifungal agents [11].

*Botrytis cinerea*, a necrotrophic phytopathogenic fungus and the causal agent of gray mold, has a wide host range and causes damage both pre-harvest and during storage [12]. *Colletotrichum* species, which are hemibiotrophic fungi, are responsible for anthracnose diseases in a wide range of woody and herbaceous plants [13]. *C. salicis*, belonging to the *C. acutatum* species complex, is associated with anthracnose of woody hosts [14] and fruit rot in crops such as peppers and tomatoes [15].

Growing environmental and regulatory pressure to reduce synthetic fungicide use has intensified interest in biological control strategies. Soil microorganisms with antifungal properties are increasingly considered sustainable alternatives in integrated plant protection systems. These microorganisms suppress phytopathogens through multiple mechanisms, including antibiosis, competition for nutrients and ecological niches, secretion of lytic enzymes, and induction of plant defense responses. Among the most extensively studied and promising biocontrol agents (BCAs) are species of *Trichoderma*, *Bacillus*, and *Streptomyces*, which exhibit diverse and often complementary antifungal mechanisms [16]. *Trichoderma* species are well-documented antagonists of *Fusarium* spp. and other soil-borne pathogens. Their biocontrol activity is based on three principal mechanisms: mycoparasitism, antibiosis, and competition for nutrients and ecological niches [17,18]. During mycoparasitism, *Trichoderma* attaches to pathogen hyphae, forms appressorium-like structures, and secretes hydrolytic enzymes such as chitinases and β-1,3-glucanases, leading to cell wall degradation and growth inhibition [19,20]. In addition, *T. asperellum* produces secondary metabolites with antifungal activity and can induce systemic resistance in plants, thereby enhancing host tolerance to infection [21]. Commercially available products for agriculture already contain various *Trichoderma* species, including *T. asperellum* [22].

Members of the genus *Bacillus* are Gram-positive, endospore-forming bacteria widely distributed in soil environments and known for their stability and adaptability [23]. Their antifungal activity is mainly due to the production of various antifungal volatile and water-soluble compounds, including cyclic lipopeptides such as iturin, surfactin, and fengycin, which disrupt fungal cell membranes and cause leakage of cell contents [24,25]. Additional mechanisms include secretion of lytic enzymes, siderophore-mediated iron competition, and induction of plant defense-related genes [26,27]. Experimental studies have demonstrated significant inhibition of *Fusarium* growth and sporulation by various *Bacillus* strains. *Bacillus subtilis* is a model organism and one of the most widely used species in biotechnology. The bioactive metabolites produced by *B. subtilis* have been classified into five groups: nonribosomal peptides, polyketides, ribosomal peptides, hybrid compounds, and volatile metabolites [28,29].

Actinobacteria of the genus *Streptomyces* are dominant components of soil microbial communities and prolific producers of bioactive secondary metabolites. They account for a substantial proportion of clinically and agriculturally relevant antibiotics [30,31]. In agricultural systems, *Streptomyces* spp. suppress phytopathogenic fungi through competition, antibiosis, emission of volatile compounds, and secretion of cell wall-degrading enzymes [32,33,34]. The two primary classes of secondary metabolites produced by *Streptomyces* are nonribosomal peptides and polyketides [35]. Several isolates have demonstrated strong antagonistic activity against toxigenic *Fusarium* species and the potential to reduce mycotoxin accumulation [36].

In general, the literature indicates that *Trichoderma*, *Bacillus*, and *Streptomyces* species possess complementary antifungal mechanisms, including enzymatic degradation of fungal cell walls, membrane disruption, and competition for nutrients. Despite this, the effectiveness of these microorganisms is often strain-dependent and varies depending on the target pathogen species. Comparative studies evaluating multiple microbial groups against diverse phytopathogens, particularly using locally isolated strains, remain limited.

Given the diversity of mechanisms employed by microbial biocontrol agents, the combined evaluation of representatives from different taxonomic groups provides a more comprehensive understanding of their antifungal potential. *Trichoderma*, *Bacillus*, and *Streptomyces* species were selected in this study, as they represent three of the most widely studied and functionally distinct groups of BCAs, encompassing fungal, bacterial, and actinobacterial antagonists. These groups differ in their modes of action, including mycoparasitism, production of diffusible and volatile metabolites, and competition for ecological niches.

A comparative assessment of these microorganisms under identical experimental conditions allows for the identification of the most effective strains and provides insight into their relative performance against different phytopathogenic fungi. In addition, the use of locally isolated strains from the Microbial Strain Collection of Latvia (MSCL) enables the evaluation of their potential applicability under regional agroecological conditions.

The aim of this study was to evaluate and compare the antifungal activity of selected *Streptomyces*, *Bacillus*, and *Trichoderma asperellum* strains, including isolates from the Microbial Strain Collection of Latvia (MSCL), against phytopathogenic fungi and to assess their potential as biocontrol agents under standardized *in vitro* conditions.

## 2. Materials and Methods

### 2.1. Microbial Strains

The antifungal activity of 14 selected microbial strains was evaluated against 16 strains of phytopathogenic fungi (Table 1). All strains used in this study were obtained from the Microbial Strain Collection of Latvia (MSCL) and maintained under standardized laboratory conditions. Certain strains, including *Trichoderma asperellum* MSCL 309 and *Bacillus stercoris* MSCL 897, were originally isolated from commercial products before being deposited in the MSCL.

### 2.2. Culture Media and Growth Conditions

Fungi were cultured on Malt Extract Agar (MEA, Millipore, Bangalore, India), while bacterial strains were cultured on R2A agar (Millipore, Darmstadt, Germany). *B. subtilis* and *B. stercoris* were subcultured 3–4 days prior to testing to ensure active growth. Fungal cultures and *Streptomyces* were incubated for 7 days at 20–22 °C before antifungal assays. All experiments were performed using three independent biological replicates. Each treatment within an experiment was conducted in triplicate, resulting in a total of nine measurements per treatment. Control plates without antagonist microorganisms were included in all assays.

### 2.3. Dual-Culture Assay

The antifungal activity of antagonist strains was evaluated using a dual-culture assay. The following antagonistic microorganisms were tested: *Trichoderma asperellum* MSCL 309 and ten strains of *Streptomyces* spp. (MSCL 346, 349, 350, 351, 354, 355, 415, 420, 422, and 424). Agar discs (0.7 cm diameter) from actively growing margins of 5–7-day-old fungal and antagonist cultures were placed 4 cm apart on MEA plates. Plates were incubated at 20–22 °C for 7 days. Control plates were prepared by inoculating only the phytopathogenic fungus under identical conditions without the antagonist strain. Antifungal activity was quantified by measuring fungal colony diameter and calculating the growth inhibition percentage: (colony diameter in control − colony diameter in treatment) × 100/colony diameter in control [37]. Colony diameter was measured along two perpendicular axes, and the average value was used for further calculations. Each fungus–antagonist combination was performed in triplicate.

### 2.4. Preparation of Microbial Suspensions

*Fusarium* spp. suspensions were prepared from 7-day-old cultures grown on MEA plates. Fungal spores and mycelium were gently scraped from the agar surface and suspended in sterile distilled water. The suspension was homogenized by vortexing and adjusted to an optical density (OD540) of 0.16. *Bacillus* spp. suspensions were prepared from overnight cultures grown on R2A agar. Bacterial cells were collected and suspended in sterile distilled water, followed by adjustment to an optical density of 0.16 at 540 OD, corresponding approximately to 10^7^–10^8^ CFU/mL.

### 2.5. Agar Well Diffusion Assay

The antifungal activity of *Bacillus* spp. strains was evaluated using an agar well diffusion assay. Prepared *Fusarium* suspensions (0.16 at OD540) were uniformly spread on MEA plates using a sterile spreader. Wells (0.7 cm in diameter) were created in the agar and filled with 70 µL of *Bacillus* suspensions (0.16 at OD540). Control wells were filled with sterile distilled water to assess baseline fungal growth. Plates were incubated at 20–22 °C for 7 days. Antifungal activity was expressed as the inhibition zone diameter. It should be noted that live bacterial suspensions were used in this assay rather than cell-free supernatants. Therefore, the observed inhibition reflects the combined effect of bacterial growth and the production of diffusible antimicrobial compounds under the experimental conditions.

### 2.6. Assessment of Volatile and Soluble Metabolites of T. asperellum

To determine the contribution of volatile organic compounds (VOCs) produced by *T. asperellum*, a double-plate assay was performed according to Sipiczki et al. (2023) [38]. *T. asperellum* and the pathogen were inoculated on the surface of agar plates in separate Petri dishes. The lids were removed, and the bottom halves of the dishes were placed facing each other so that the inoculated agar surfaces were aligned. The paired dishes were sealed with several layers of Parafilm to prevent the escape of VOCs. Fungal growth inhibition was evaluated after 7 days of incubation at 20–22 °C. Control plates were prepared in the same manner, but without inoculation of the antagonist strain. The pathogen plate was paired with a sterile agar plate, allowing for the assessment of fungal growth under sealed conditions in the absence of volatile organic compounds.

The effect of soluble/diffusible metabolites of *T. asperellum* was evaluated using a cellophane overlay method [38]. *T. asperellum* was cultivated on MEA plates covered with sterile cellophane (Bright Ideas Crafts, Bury St. Edmunds, UK) for 7 days. After incubation, the fungal biomass together with the cellophane was removed, leaving the agar medium enriched with soluble/diffusible metabolites released by *T. asperellum* during growth. Colony diameter was measured after 48 h to 72 h at 20–22 °C, and growth inhibition percentage was calculated relative to untreated controls.

### 2.7. Statistical Analysis

Statistical analyses were conducted using RStudio (version 2024.12.1 + 563). Data were tested for normality prior to analysis. One-way ANOVA was applied to evaluate the effect of antagonist strain on fungal growth inhibition, while two-way ANOVA was used to assess interactions between antagonist strains and fungal species. Duncan’s multiple range test was used for post hoc comparisons. Differences were considered statistically significant at *p* < 0.05.

## 3. Results

### 3.1. Antifungal Activity of Trichoderma asperellum Against Fusarium spp.

#### 3.1.1. Dual-Culture Test

**Figure 1:**
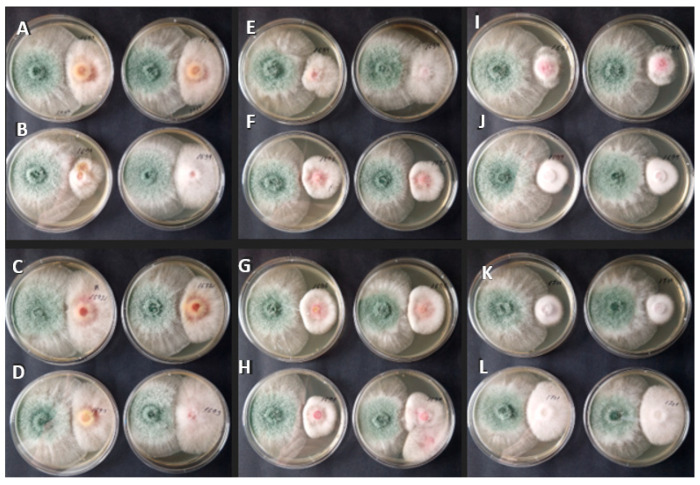
Representative images of dual-culture assays showing the interaction between *Trichoderma asperellum* MSCL 309 (left side) and (**A**) *Fusarium culmorum* MSCL 1690, (**B**) *F. graminearum* MSCL 1691, (**C**) *F. culmorum* MSCL 1692, (**D**) *F. culmorum* MSCL 1693, (**E**) *F. graminearum* MSCL 1694, (**F**) *F. sporotrichioides* MSCL 1695, (**G**) *F. oxysporum* MSCL 1696, (**H**) *F. sporotrichioides* MSCL 1697, (**I**) *F. tricinctum* MSCL 1698, (**J**) *F. oxysporum* MSCL 1699, (**K**) *F. oxysporum* MSCL 1700, and (**L**) *F. poae* MSCL 1701 (right side) after seven days of incubation at 20–22 °C. Images illustrate the extent of growth inhibition observed under *in vitro* conditions. Two independent replicates are shown.

The antifungal activity of *T. asperellum* MSCL 309 against twelve phytopathogenic *Fusarium* isolates obtained from oats in Latvia was assessed using the dual-culture assay. After seven days of incubation, growth inhibition was observed for all tested isolates. The growth inhibition ranged from 44.6% to 78.4% (Table 2, Figure 1), indicating substantial variability in isolate susceptibility. The highest inhibition, 71.0–78.4%, was observed for both strains of *F. sporotrichioides* (MSCL 1697 and MSCL 1695), two strains of *F. oxysporum* (MSCL 1696 and MSCL 1700), *F. culmorum* MSCL 1693, and *F. poae* MSCL 1701. Both strains of *F. graminearum* (MSCL 1691 and MSCL 1694) exhibited significantly (*p* < 0.05) lower sensitivity. The consistent inhibition across all isolates indicates that *T. asperellum* possesses broad-spectrum antifungal potential against *Fusarium* spp., although the degree of inhibition is species- and/or isolate-dependent.

#### 3.1.2. Contribution of Volatile Organic Compounds

The contribution of volatile organic compounds (VOCs) produced by *T. asperellum* to antifungal activity was evaluated using a sealed plate assay. After 72 h of incubation, no statistically significant differences (*p* > 0.05) in colony diameter were observed between treated and control plates for any of the tested *Fusarium* isolates. Although minor variations in radial growth were visually detectable in some cases, these differences were inconsistent and not statistically supported.

These results indicate that VOC-mediated inhibition was negligible under the experimental conditions used. The lack of a significant effect suggests that either VOCs were not produced in sufficient concentrations or that they did not exhibit strong antifungal activity against the tested isolates in this assay system.

#### 3.1.3. Contribution of Soluble Metabolites

**Figure 2:**
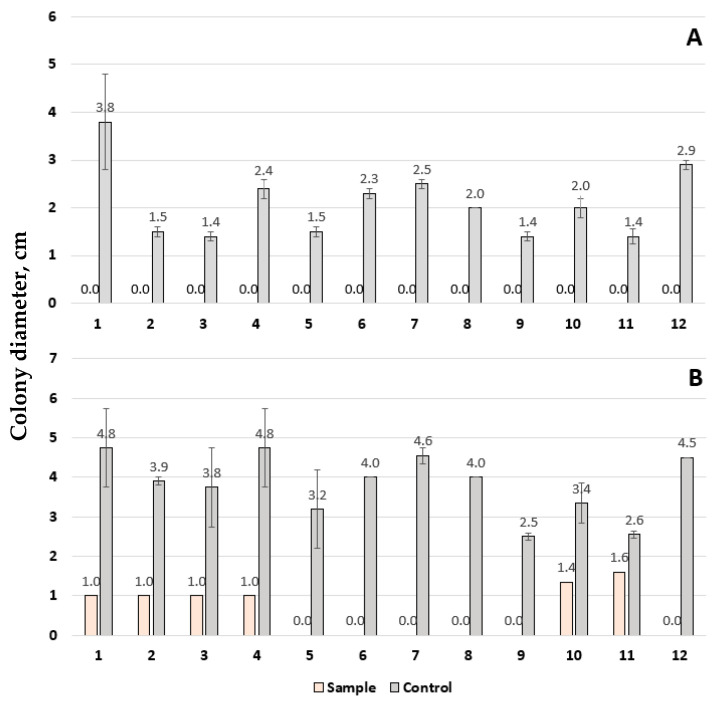
Colony diameter (cm ± SD) of *Fusarium* isolates grown on control medium and medium pretreated with soluble metabolites of *Trichoderma asperellum* MSCL 309 after 48 h (**A**) and 72 h (**B**) of incubation. Data represent the mean values of three independent experiments. Strain numbering corresponds to the following: 1. *Fusarium culmorum* MSCL 1690; 2. *F. graminearum* MSCL 1691; 3. *F. culmorum* MSCL 1692; 4. *F. culmorum* MSCL 1693; 5. *F. graminearum* MSCL 1694; 6. *F. sporotrichioides* MSCL 1695; 7. *F. oxysporum* MSCL 1696; 8. *F. sporotrichioides* MSCL 1697; 9. *F. tricinctum* MSCL 1698; 10. *F. oxysporum* MSCL 1699; 11. *F. oxysporum* MSCL 1700; 12. *F. poae* MSCL 1701.

**Figure 3:**
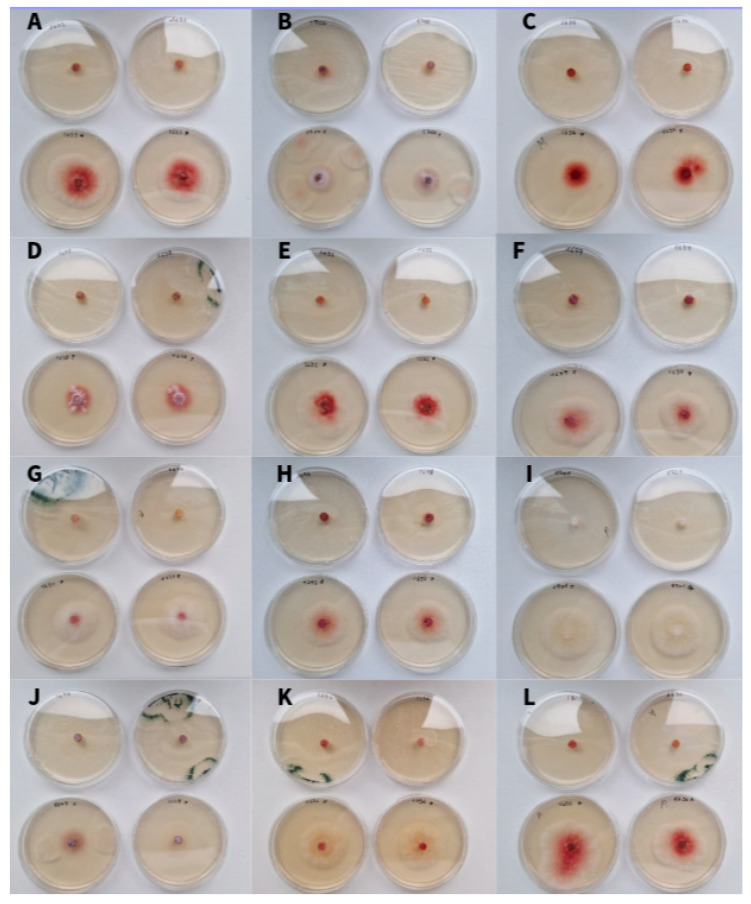
Colony diameter (cm ± SD) of *Fusarium* isolates grown on control medium and medium pretreated with soluble metabolites of *Trichoderma asperellum* MSCL 309 after 48 h (the top two images next to each letter) and 72 h (the two images below each letter) of incubation. Data represent the mean values of three independent experiments. Strain numbering corresponds to the following: (**A**)—*Fusarium culmorum* MSCL 1690, (**B**)—*F. graminearum* MSCL 1691, (**C**)—*F. culmorum* MSCL 1692, (**D**)—*F. culmorum* MSCL 1693, (**E**)—*F. graminearum* MSCL 1694, (**F**)—*F. sporotrichioides* MSCL 1695, (**G**)—*F. oxysporum* MSCL 1696, (**H**)—*F. sporotrichioides* MSCL 1697, (**I**)—*F. tricinctum* MSCL 1698, (**J**)—*F. oxysporum* MSCL 1699, (**K**)—*F. oxysporum* MSCL 1700, and (**L**)—*F. poae* MSCL 1701. Two replicates are shown.

In contrast to VOCs, soluble metabolites produced by *Trichoderma* showed strong antifungal activity. After 48 h of incubation, colony diameters on control plates ranged from 1.4 to 3.8 cm, while plates pretreated with soluble *T. asperellum* metabolites showed complete growth inhibition (Figure 2A). After 72 h, the diameter of the control *Fusarium* colonies had increased 1.5–2-fold (Figure 2B). However, complete inhibition was still observed for isolates from *F. graminearum* MSCL 1694, both strains of *F. sporotrichioides* (MSCL 1695 and MSCL 1697), *F. oxysporum* MSCL 1696, *F. tricinctum* MSCL 1698, and *F. poae* MSCL 1701 on pretreated plates. The other isolates also had significantly reduced colony diameters (1.0–1.6 cm), consistent with significant growth inhibition. These results demonstrate that soluble metabolites are the primary contributors to the antifungal activity of *T. asperellum* at least under *in vitro* conditions (Table 2, Figure 2 and Figure 3).

### 3.2. Antifungal Activity of Bacillus Species Against Fusarium spp.

**Figure 4:**
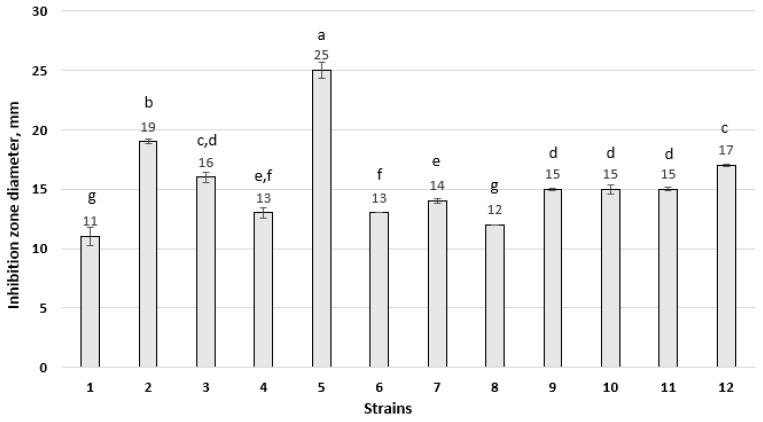
Diameter of the zone of inhibition of *Fusarium* (mm ± SD) after seven days of cultivation in an agar well assay with *B. subtilis* MSCL 1441. Strain transcription: 1. *Fusarium culmorum* MSCL 1690; 2. *F. graminearum* MSCL 1691; 3. *F. culmorum* MSCL 1692; 4. *F. culmorum* MSCL 1693; 5. *F. graminearum* MSCL 1694; 6. *F. sporotrichioides* MSCL 1695; 7. *F. oxysporum* MSCL 1696; 8. *F. sporotrichioides* MSCL 1697; 9. *F. tricinctum* MSCL 1698; 10. *F. oxysporum* MSCL 1699; 11. *F. oxysporum* MSCL 1700; 12. *F. poae* MSCL 1701. Numbers with different letters above the columns are significantly different (*p* < 0.05).

**Figure 5:**
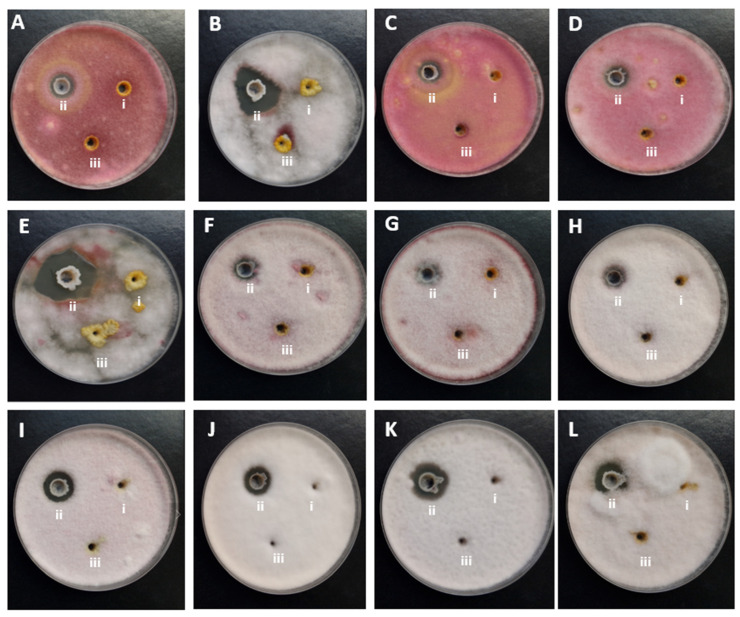
Effect of three *Bacillus* strains plated in agar wells on the growth of *Fusarium* spp. after seven days of cultivation. i—*B. stercoris* MSCL 897, ii—*B. subtilis* MSCL 1441, iii—*B. subtilis* MSCL 49. (**A**) *Fusarium culmorum* MSCL 1690, (**B**) *F. graminearum* MSCL 1691, (**C**) *F. culmorum* MSCL 1692, (**D**) *F. culmorum* MSCL 1693, (**E**) *F. graminearum* MSCL 1694, (**F**) *F. sporotrichioides* MSCL 1695, (**G**) *F. oxysporum* MSCL 1696, (**H**) *F. sporotrichioides* MSCL 1697, (**I**) *F. tricinctum* MSCL 1698, (**J**) *F. oxysporum* MSCL 1699, (**K**) *F. oxysporum* MSCL 1700, and (**L**) *F. poae* MSCL 1701. One of three replicates is shown.

An experiment was conducted using two *B. subtilis* strains, MSCL 49 and MSCL 1441, and *B. stercoris* MSCL 897 to evaluate the antifungal activity of bacterial suspensions against twelve *Fusarium* spp. Isolates. Antifungal activity was observed in only one strain, *B. subtilis* 1441 (Figure 4 and Figure 5). The largest diameter of the zone of inhibition was observed for both *F. graminearum* strains, MSCL 1694 and MSCL 1691, at 25 mm and 19 mm, respectively. The lowest inhibition was observed for *F. culmorum* MSCL 1690 (11 mm) and *F. sporotrichioides* MSCL 1697 (12 mm).

### 3.3. Antifungal Activity of Streptomyces spp. Against Selected Phytopathogenic Fungi

**Figure 6:**
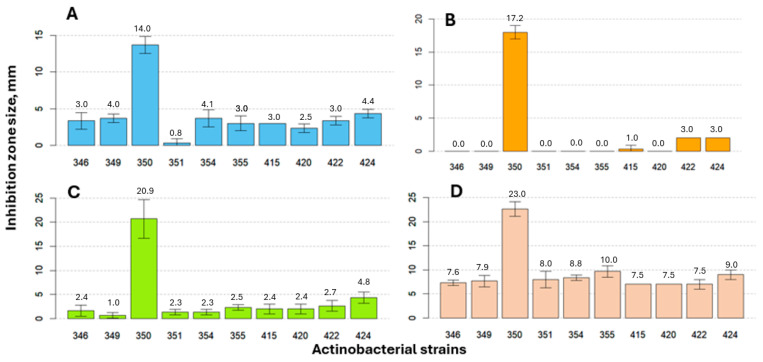
Inhibition zone size (mm ± SD) of *Fusarium oxysporum* (**A**), *F. graminearum* (**B**), *Botrytis cinerea* (**C**), and *Colletotrichum salicis* (**D**) dependent on the opposite growing actinobacteria *Streptomyces griseus* MSCL 346, *Streptomyces* sp. MSCL 349, *S. venezuelae* MSCL 350, *S. griseus* MSCL 351, *S. sylvae* MSCL 354, *Streptomyces* sp. MSCL 355, *Streptomyces* sp. MSCL 415, *S. anthocyanicus* MSCL 420, *S. griseus* MSCL 422, and *S. griseus* MSCL 424 in three replicates after seven days of incubation.

Ten *Streptomyces* strains were evaluated against four phytopathogenic fungi using the dual-culture assay. Antifungal activity varied markedly depending on both antagonist strain and fungal species (Figure 6). Across all tested *Streptomyces* strains, *S. venezuelae* MSCL 350 exhibited the highest antifungal activity against all pathogens, highlighting its superior antagonistic capacity. For *F. oxysporum*, the maximum inhibition zone diameter was 13.7 mm when paired with *S. venezuelae*. Other *Streptomyces* strains showed considerably weaker inhibition, ranging from 0.3 mm to 4.3 mm. *F. graminearum* demonstrated relatively high resistance, and significant inhibition was observed only in *S. venezuelae* (18.0 mm), while inhibition was minimal or absent for most other strains.

*Botrytis cinerea* showed moderate sensitivity. The strongest inhibition was again observed with *S. venezuelae* (20.7 mm), while the other strains produced inhibition zone diameters ranging from 1.3 mm to 4.3 mm.

The most sensitive species tested was *Colletotrichum salicis*. The diameter of the inhibition zone for *S. venezuelae* reached 22.7 mm, and other *Streptomyces* strains also caused significant inhibition (7.0–9.7 mm). This suggests a greater sensitivity of *C. salicis* to actinobacterial antagonism compared to *F. graminearum* and *B. cinerea*.

Two-way ANOVA supports the conclusion that antifungal activity depends not only on the antagonist strain but also on the intrinsic properties of the phytopathogenic fungus. These findings emphasize the importance of strain selection in BCA applications and suggest that *S. venezuelae* MSCL 350 represents a particularly promising candidate for further development.

## 4. Discussion

The present study demonstrates that antifungal activity is strongly dependent on both the antagonist strain and the phytopathogen species and strain. Among the tested microorganisms, *Trichoderma asperellum* MSCL 309 and *Streptomyces venezuelae* MSCL 350 exhibited the highest and most consistent antifungal activity, whereas the investigated *Bacillus* spp. showed comparatively moderate effects.

### 4.1. Antifungal Mechanisms of Trichoderma asperellum

The dual-culture assay revealed broad-spectrum inhibition of all tested *Fusarium* isolates by *T. asperellum*, supporting previous reports describing *Trichoderma* spp. as effective antagonists of soil-borne phytopathogens [17,18]. Experiments show that of the *Fusarium* species studied, *F. graminearum* is relatively difficult to control (Table 1). The observed isolate-dependent growth inhibition percentage is consistent with documented variability in susceptibility among *Fusarium* species and strains [1,2].

The absence of statistically significant inhibition in VOC assay suggests that VOCs played a limited role under the applied *in vitro* conditions. Although *Trichoderma* spp., including *T. asperellum*, are known to produce VOCs with antifungal properties [39], their efficacy may depend on environmental conditions and metabolite concentration [20]. The present findings suggest that the antagonism mediated by VOCs was negligible compared to other metabolites.

In contrast, soluble metabolites demonstrated strong antifungal activity, including the complete inhibition of several isolates. This observation aligns with established mechanisms of *Trichoderma* antagonism, which involve the secretion of hydrolytic enzymes (chitinases and β-1,3-glucanases) and secondary metabolites that degrade fungal cell walls or disrupt membrane integrity [19,20]. Such enzymatic and metabolite-driven inhibition likely represents the primary mechanism of antifungal activity observed in this study.

### 4.2. Strain-Specific Antifungal Activity of Bacillus spp.

*B. subtilis* MSCL 1441 showed significant antifungal activity, compared to *B. subtilis* MSCL 49 and *B. stercoris* MSCL 897, confirming the strain-dependent nature of the antagonism. *Bacillus* spp. are widely recognized for producing cyclic lipopeptides such as iturins, surfactins, and fengycins, which disrupt fungal cell membranes [24,25]. It has been found that both *B. subtilis* MSCL 1441 and *B. stercoris* MSCL 897 synthesize surfactins and fengycins, but not iturins [40]; however, the antagonistic activity differed significantly, indicating the involvement of additional factors.

These differences may be related to variation in the quantity of metabolite production, regulation of biosynthetic pathways, or the presence of other bioactive compounds. In addition to lipopeptide production, *Bacillus* spp. may contribute to pathogen inhibition through siderophore-mediated iron competition and induction of plant defense responses [26,27]. However, under the strictly *in vitro* conditions of this study, the dominant mechanism was most likely direct metabolite-mediated inhibition.

*B. subtilis* MSCL 1441 stood out among all tested antagonists in its ability to strongly inhibit *F. graminearum* (Figure 3), a species that was relatively resistant to other microorganisms examined in this study. This finding highlights the potential of specific *Bacillus* strains to target otherwise difficult-to-control phytopathogens.

*B. stercoris* belongs to the “*subtilis* group” and “*subtilis* subgroup” [41] and was originally identified as *B. subtilis* subsp. *stercoris*. Based on genomic analysis, it was later elevated to species level [42]. Despite this taxonomic proximity to *B. subtilis, B. stercoris* MSCL 897 did not exhibit antifungal activity under the conditions tested, indicating that taxonomic classification alone is not sufficient to predict biocontrol potential and emphasizing the importance of strain-level evaluation.

In the present study, the antifungal activity of *Bacillus* strains was evaluated using whole-cell suspensions, reflecting their overall antagonistic potential under *in vitro* conditions. In contrast, a more detailed mechanistic analysis was performed only for *Trichoderma asperellum*, which was selected as a model organism for investigating the contribution of volatile and soluble metabolites.

Although *Bacillus* spp. are well known to produce antifungal compounds such as cyclic lipopeptides, including surfactins and fengycins, the specific contribution of these metabolites was not directly assessed in this study. Such analyses would require the use of cell-free supernatants or purified compounds and were beyond the scope of the present work. Further studies are needed to characterize the role of individual metabolites in the observed antifungal activity.

### 4.3. Superior Antagonistic Potential of Streptomyces venezuelae

The dual-culture assay demonstrated that antifungal activity among *Streptomyces* spp. was species- or strain-dependent, with *S. venezuelae* MSCL 350 producing the largest inhibition zone diameters. This is consistent with the well-documented capacity of *Streptomyces* spp. to synthesize diverse bioactive secondary metabolites, including antibiotics with antifungal properties [30,31]. The strong antifungal activity observed for *S. venezuelae* MSCL 350 suggests the production of extracellular bioactive metabolites. Similar findings have been reported for other *Streptomyces* strains capable of suppressing phytopathogenic fungi through antibiotic production [33,34]. The variability in antifungal activity among the tested strains further highlights the importance of strain-specific properties in determining biocontrol potential.

Soil bacterium *S. venezuelae* is known as an antibiotic (chloramphenicol, jadomycin, and pikromycins) producer and differs from many other *Streptomyces* in its ability to complete its entire life cycle in liquid culture [43]. It is possible that this is what allowed this bacterium to display properties of its extracellular metabolites that were not possible for other *Streptomyces* under liquid cultivation. The expression of secondary metabolite biosynthesis gene clusters in *Streptomyces* spp. is associated with the complex morphological differentiation of the bacteria during the transition from vegetative to aerial mycelium and subsequent sporulation; therefore, these genes are called silent or cryptic, and under laboratory conditions, they are usually weakly expressed [44].

Species-dependent sensitivity was evident in this study. *Colletotrichum salicis* showed the highest sensitivity (Figure 5D), while *Fusarium graminearum* showed relatively higher resistance (Figure 5B), as in the case of *T. asperellum* (Table 1). Such differences may be linked to intrinsic pathogenicity mechanisms, cell wall architecture, and adaptive stress responses [6,7].

The consistent activity of *S. venezuelae* MSCL 350 against multiple phytopathogenic fungi suggests its potential as a broad-spectrum BCA.

## 5. Conclusions

This study demonstrates that antifungal activity against phytopathogenic fungi is strongly strain-dependent and varies among microbial groups. Among the tested antagonists, *Trichoderma asperellum* MSCL 309 and *Streptomyces venezuelae* MSCL 350 exhibited the most consistent and broad-spectrum antifungal activity, while *Bacillus subtilis* MSCL 1441 showed strong but more specific inhibition, particularly against *Fusarium graminearum*.

The results indicate that soluble metabolites are the primary contributors to the antifungal activity of *T. asperellum* under *in vitro* conditions, whereas volatile compounds had negligible effects. The observed variability in pathogen sensitivity highlights the importance of targeted strain selection for effective biocontrol strategies.

Overall, the identified strains represent promising candidates for the development of environmentally friendly biocontrol agents. Future research should focus on the characterization of antifungal metabolites and the validation of their efficacy under greenhouse and field conditions.

## Figures and Tables

**Table 1:** Microorganisms used in the study.

Species	MSCL Number	Origin	Type of Identification
Antagonists			
*Bacillus subtilis*	1441	Lupine soil, Latvia	biochemical
*Bacillus subtilis*	49	Unknown	biochemical
*Bacillus stercoris*	897	Commercial cleaner, Latvia	rRNA gene
*Streptomyces anthocyanicus*	420	Garden soil, Latvia	ANI
*Streptomyces griseus*	346	Garden soil, Latvia	rRNA gene, ANI
*Streptomyces griseus*	351	Garden soil, Latvia	rRNA gene, ANI
*Streptomyces griseus*	422	Garden soil, Latvia	ANI
*Streptomyces griseus*	424	Garden soil, Latvia	ANI
*Streptomyces silvae*	354	Garden soil, Latvia	rRNA gene
*Streptomyces* sp.	355	Garden soil, Latvia	rRNA gene
*Streptomyces* sp.	415	Garden soil, Latvia	micromorphological
*Streptomyces* sp.	349	Garden soil, Latvia	rRNA gene
*Streptomyces venezuelae*	350	Garden soil, Latvia	ANI
*Trichoderma asperellum*	309	Commercial preparation, Latvia	ITS
Pathogens			
*Botrytis cinerea*	433	Liver paste, Sweden	unknown
*Colletotrichum salicis*	850	Rhododendron leaves, Riga	ITS, LSU, TUB2, ACT, CHS-1, GAPDH, HIS3
*Fusarium oxysporum*	259	Unknown	unknown
*Fusarium graminearum*	435	Oat, Sweden	unknown
*Fusarium culmorum*	1690	*Avena sativa*, Latvia	micromorphological
*Fusarium culmorum*	1692	*Avena sativa*, Latvia	ITS, TEF
*Fusarium culmorum*	1693	*Avena sativa*, Latvia	ITS, TEF
*Fusarium graminearum*	1691	*Avena sativa*, Latvia	ITS, TEF
*Fusarium graminearum*	1694	*Avena sativa*, Latvia	ITS, TEF
*Fusarium oxysporum*	1696	*Avena sativa*, Latvia	micromorphological
*Fusarium oxysporum*	1699	*Avena sativa*, Latvia	ITS, TEF
*Fusarium oxysporum*	1700	*Avena sativa*, Latvia	ITS, TEF
*Fusarium poae*	1701	*Avena sativa*, Latvia	ITS, TEF
*Fusarium sporotrichioides*	1695	*Avena sativa*, Latvia	ITS, TEF
*Fusarium sporotrichioides*	1697	*Avena sativa*, Latvia	ITS, TEF
*Fusarium tricinctum*	1698	*Avena sativa,* Latvia	micromorphological

**Table 2:** Inhibitory effect of *T. asperellum* against twelve *Fusarium* strains. Numbers in a column followed by different letters are significantly different (*p* < 0.05).

*Fusarium* Strains	Percentage of Inhibition Under Dual-Culture Conditions
*Fusarium culmorum* MSCL 1690	49.1 ± 4.4 ^c^
*Fusarium graminearum* MSCL 1691	44.6 ± 10.5 ^c^
*Fusarium culmorum* MSCL 1692	46.7 ± 5.8 ^c^
*Fusarium culmorum* MSCL 1693	71.4 ± 4.5 ^a^
*Fusarium graminearum* MSCL 1694	48.9 ± 9.4 ^b,c^
*Fusarium sporotrichioides* MSCL 1695	71.0 ± 3.9 ^a^
*Fusarium oxysporum* MSCL 1696	74.4 ± 5.6 ^a^
*Fusarium sporotrichioides* MSCL 1697	78.4 ± 7.0 ^a^
*Fusarium tricinctum* MSCL 1698	56.7 ± 3.3 ^b^
*Fusarium oxysporum* MSCL 1699	56.7 ± 6.6 ^b^
*Fusarium oxysporum* MSCL 1700	74.1 ± 3.5 ^a^
*Fusarium poae* MSCL 1701	71.0 ± 4.1 ^a^

## Data Availability

The original contributions presented in this study are included in the article. Further inquiries can be directed to the corresponding author.

## Abbreviations

The following abbreviations are used in this manuscript:

BCA	biocontrol agent
FHB	*Fusarium* head blight
MSCL	Microbial Strain Collection of Latvia
VOCs	volatile organic compounds
